# Framing Effects of Cognitive Behavioural Therapy for Depression on Perceptions of Believability, Acceptability, and Credibility

**DOI:** 10.3390/ijerph20146330

**Published:** 2023-07-09

**Authors:** Saba Salimuddin, Shadi Beshai, Adam Iskric, Lisa Watson

**Affiliations:** 1 Department of Psychology, University of Regina, Regina, SK S4S 0A2, Canada; 2 Department of Psychology, Hofstra University, Hempstead, NY 11549, USA; 3 Faculty of Business, Athabasca University, Athabasca, AB T9S 3A3, Canada

**Keywords:** health communication, mental health literacy, tailored messaging

## Abstract

While CBT is an effective treatment for depression, uptake can be low. This is largely due to attitudinal barriers. Accordingly, the goals of the current investigation were to (a) tailor and develop persuasive psychoeducational materials to match dominant cultural beliefs about the causes of depression and (b) examine the effectiveness of tailored CBT descriptions in improving CBT perceptions. We examined the believability of CBT mechanisms by invoking commonly endorsed etiological models of depression and investigated whether tailoring CBT descriptions to match etiological beliefs about depression influences perceptions of CBT. Participants were recruited using TurkPrime. In Study 1, participants (*n* = 425) read a CBT description that was generic or framed to match an etiological model of depression (biological, stress/environmental, or relationship/interpersonal). The participants indicated believability of each model as adopted by CBT. In study 2, the participants (*n* = 449) selected what they believed was the most important cause of depression. Subsequently, the participants were randomised to receive either a CBT description tailored to their endorsed model or a generic CBT description, and they provided ratings for CBT’s acceptability, credibility, and expectancy. In Study 1, the believability of biological CBT mechanisms was low across conditions, but participants reported greater believability when receiving a biological description than when receiving other mechanistic descriptions. Participants who received the stress- and relationship-focused descriptions did not rate the respective models as more believable than those who received a generic description. In study 2, there were no differences in the perceptions of acceptability, credibility and expectancy between participants who received a tailored description and those who received a generic description. Our findings suggest that CBT is believed to be a psychologically appropriate treatment; however, the believability of biological mechanisms is improved by presenting a biology-focused description.

## 1. Introduction

Depression is among the leading causes of disability around the world [1,2]. Researchers have consistently demonstrated that depression is associated with significant costs on personal and societal levels [3,4]. Cognitive behavioural therapy (CBT) is a first-line treatment for depression with extensive support as an effective intervention for the condition [5]. CBT involves learning to identify and challenge maladaptive thinking and behavioural patterns that are thought to be contributing to current problems. Unfortunately, very few individuals who would benefit from mental health care go on to initiate or engage with appropriate treatments, let alone CBT [6]. Moreover, meta-analyses examining dropout rates among those who initiate CBT reveal high dropout rates, which appear to be especially pronounced among patients with depression [7,8,9,10]. Several factors explain the poor uptake of and engagement in CBT and other treatments for depression, including systemic and logistical barriers to treatment (e.g., financial constraints; time commitment; physical distance). Importantly, there are also emotional and attitudinal barriers that slow uptake and engagement in depression treatments, with these barriers being more readily modifiable than their systemic counters [11,12]. These attitudinal barriers include stigma around seeking treatment and perceived ineffectiveness or inappropriateness of a target treatment for a given concern [13,14]. Accordingly, examining the nature and malleability of perceptions relating to CBT’s effectiveness, credibility, and acceptability as a treatment for depression may offer a cost-effective path to improving CBT initiation and engagement metrics. 

Patient perceptions of mental health treatment are associated with several important outcomes. Three of these treatment perception factors—acceptability, credibility, and expectancy—are consistently associated with uptake and engagement. Acceptability is defined as perceptions of the fairness, non-intrusiveness, and appropriateness of a treatment for a given problem [15,16]. Treatment credibility is related to how logical and convincing a treatment seems, while expectancy is the perception of how much improvement is expected with a given treatment [17]. Treatment credibility and expectancy beliefs predict engagement with treatment [18], the quality of the therapeutic alliance [19], dropout from treatment [20], and outcomes such as symptom reduction [21,22]. Encouragingly, the recent literature on treatment perceptions of CBT has suggested that these beliefs are malleable when brief, low-cost psychoeducational interventions are used. For instance, Soucy et al. [23] found that both treatment seekers and non-seekers rated internet-delivered CBT more positively after viewing a short video that described CBT as an intervention for depression and anxiety. Furthermore, Schofield et al. [24] found that knowledge and perceptions of CBT for anxiety improved following exposure to a webpage with a brief description of the treatment. Similarly, Beshai et al. [25] found that participants’ perceptions of treatment credibility and expectancy improved significantly after reading a brief evidence-based description of CBT for depression. 

There are other attitudinal and literacy-related factors that associate meaningfully with CBT acceptability and engagement metrics. Etiological models of disorders or personal explanations of how and why disorders develop and expectations about recovery processes appear to moderate perceptions of CBT and associate with several other treatment-related outcomes [25,26,27]. Causal attributions for the development of mental illness predict help-seeking behaviours [28], treatment compliance [29], treatment preference, and the characteristics of a preferred healthcare provider [30]. 

Importantly, beliefs about causes of depression appear to interact with treatment perception factors to predict preferences and other outcomes. Schweizer et al. [31] found that patients with depression were more likely to select CBT as their preferred treatment when they believed their depression was caused by intraindividual (i.e., characterological, existential, physical, and achievement-related) factors, and a psychopharmacological treatment when they believed their depression was caused by biological factors. Further, Khalsa [32] found that patients with depression preferred psychotherapy over medication when they endorsed childhood or complex (i.e., more than one cause) causes of depression. Beshai et al. [25] found that greater endorsement of a biological model of depression was associated with lower scores for CBT’s acceptability and credibility as a treatment for depression. More recently, Watson and Beshai [27] found that community adults endorsing biological causes of depression were more likely to select medication as their preferred treatment, whereas those endorsing personality-related causes of depression were likely to select CBT as their preferred treatment. Taken together, these findings suggest that individuals’ explanatory models regarding the causes of depression appear to moderate beliefs regarding the appropriateness and logic of CBT as a treatment option for the disorder.

These converging lines of evidence suggest that to improve the uptake of and engagement in CBT, researchers and clinicians might consider tailoring the messaging around the nature and benefits of CBT so that the messaging closely matches individuals’ pre-existing explanatory models of distress. Tailored health communications refer to messages promoting health-related knowledge wherein the relevant information is modified to fit the characteristics, needs, or beliefs of a person based on a prior assessment of them. In contrast, generic health communications are not individualised in any capacity [33]. In their meta-analysis, Noar et al. [34] demonstrated that tailored messages are more effective than targeted messages (i.e., messages aimed at certain segments of the population, usually based on demographic characteristics but not individualised to one specific person) and generic messages in promoting health behaviour change. 

Models of persuasion offer a mechanistic narrative of how tailored health messaging may function to shift health attitudes. One such model is the elaboration likelihood model [35], which proposes two routes of processing persuasive information: central and peripheral. Central route processing involves active and careful engagement with the information being presented. Peripheral route processing involves little cognitive effort and relies on heuristics to process surface-level cues instead of engaging with the core argument of the message. The former results in a long-term attitude change, whereas the latter produces relatively short-lived effects. According to Petty et al. [36], tailored messages are effective tools of persuasion because they increase the likelihood of central route processing by presenting individuals with personally relevant information. This stands in contrast to generic messages, which may not be as effective in invoking active engagement with the message due to their impersonal nature. 

To our knowledge, only one study has investigated the effects of treatment descriptions incorporating specific mechanisms of change in CBT on participant perceptions of treatment. Schofield et al. [24] randomised participants to receive a description citing psychological, neurological, neuropsychological, or no mechanisms of change in CBT for anxiety. Although knowledge and perceptions of CBT improved following exposure to the descriptions, the researchers found no main effect of the description theme on CBT perceptions. Further, participants who received a neurological or neuropsychological description showed less improvement in knowledge than those who received psychological or no-mechanism descriptions. However, it is important to note that Schofield et al. measured perceptions of CBT for anxiety. The lack of studies examining perceptions of CBT for depression specifically is a notable knowledge gap; Prins et al. [37] suggested that anxiety patients tend to view psychological interventions as more helpful than other treatments, whereas depression patients tend to perceive both biological and psychological interventions as effective. 

In summary, there is a need to package treatments in a way that promotes their uptake and engagement. Perceptions of CBT for depression are meaningfully associated with treatment uptake, dropout rates, and outcomes. Further, lay explanatory models of how depression develops or is maintained moderate perceptions of treatment acceptability, credibility, and expectancy. Finally, brief psychoeducational interventions have been effective in modifying perceptions of CBT, and these brief interventions may potentially be optimised by tailoring them, as tailored health messages tend to be more effective than generic messages. The current investigation sought to extend these findings pertaining to health communication, perceptions of aetiology, and treatment perceptions, and address gaps in the literature by examining these in the context of CBT for depression. As described below, we used novel methodologies (e.g., by using simple and complex versions of descriptions) in addressing the research questions. 

### Current Investigation

The goals of the current two-study investigation were to (1) develop persuasive psychoeducational materials that match dominant cultural beliefs about the causes of depression and (2) examine the effectiveness of tailored CBT descriptions matching baseline perceptions of the causes of depression in improving perceptions of CBT’s acceptability and credibility among a sample of adults from the general population. We sought to recruit a general community sample as social factors relating to mental health and help-seeking within the broader culture (e.g., attitudes in one’s social networks) are crucial in fostering positive attitudes towards treatment and encouraging the uptake of treatment [38]. 

In Study 1, we randomised participants to receive either a generic description of CBT for depression or a description of CBT matched to biological/chemical, stress/environmental, or relationship/interpersonal causal models of depression. Participants who received the latter descriptions read either a simple version of the respective description or a complex version. The selection of description themes was guided by Watson and Beshai’s [27] findings wherein individuals from a general population sample most frequently endorsed biological/chemical (28.6%), relationship/interpersonal (20.5%), and stress/environmental (16.7%) causes of depression. After the participants read the description to which they were assigned, we measured their levels of endorsement of the three causal models of depression and the believability of CBT’s ability to disrupt the appropriate mechanism (e.g., changing brain functioning in the biology conditions) outlined in the description. 

In Study 2, we asked participants to select from a list of options which they believed to be the most likely cause of depression. We then randomly assigned half of the sample to read a tailored description of CBT that matched their selected causal mechanism, while the other half was assigned a generic description of CBT to read. We then assessed participant perceptions of CBT’s acceptability, credibility, and expectancy as a treatment for depression. 

Corresponding to the literature cited above, our hypotheses were as follows:

1. Participants receiving either one of two biology-focused descriptions will report a significantly greater believability of CBT’s ability to disrupt biological mechanisms in depression compared to those in the control condition (Study 1).
2. Participants receiving either one of two stress-focused descriptions of CBT will report a significantly greater believability of CBT’s ability to disrupt stress mechanisms in depression compared to those in the control condition (Study 1).
3. Participants receiving either one of two interpersonally focused descriptions of CBT will report a significantly greater believability of CBT’s ability to disrupt relationship-based mechanisms in depression compared to those in the control condition (Study 1).
4. Participants receiving complex descriptions will rate the matching explanatory models as significantly more believable than those receiving simple descriptions (Study 1).
5. Participants in the tailored condition will rate CBT’s acceptability, credibility, and expectancy significantly more favourably than participants in the generic condition (Study 2).

Finally, we explored the relationships between demographic factors and mechanistic believability (Study 1), as well as the relationships between demographics and perceptions of acceptability, credibility, and expectancy (Study 2). 

## 2. Study 1 Method

The procedures and hypotheses corresponding to this study were pre-registered prior to data collection (AsPredicted #18774).

### 2.1. Participants and Recruitment

An online adult community sample of 425 participants, including both treatment naïve and non-naïve participants, was recruited using TurkPrime, an online crowdsourcing platform [39]. Online crowdsourcing platforms such as TurkPrime provide access to large and diverse samples; they are popular means of sample recruitment within behaviour science research and are increasingly being adopted within clinical research [40]. To estimate the sample size, we carried out a G*Power calculation [41]. We estimated for a conservative small-medium effect, consistent with previous studies examining the effects of CBT marketing on improving consumer perceptions of the treatment [42]. The power calculation was set to a power of 90%, and a seven-group design with a multivariate analysis of variance (MANOVA) was used. The estimated appropriately powered sample size for this design was *n* = 352; however, we anticipated the removal of 15–20% inattentive responses [43] and accordingly recruited 425 participants. The eligibility criteria included (a) being aged 18 years or older; (b) fluency in English; (c) passing the included attention-check test; and (d) residing in an English-speaking country (i.e., the United States, United Kingdom, Canada, Australia, and New Zealand; see Table A1 for participant demographics). 

### 2.2. Materials

#### 2.2.1. CBT Descriptions

We adapted a brief, expert-vetted generic description of CBT for depression [25] to create the six descriptions incorporating specific causal mechanisms of depression. The descriptions provided a summary of the nature of CBT, its purpose, and how it is purported to alleviate symptoms, followed by five evidence-based advantages and disadvantages of the use of CBT for depression. Refer to the Supplementary Information for the CBT descriptions.

The descriptions provided were either (1) generic, describing CBT’s nature with only vague reference to mechanisms, or (2) framed within a (A) biological/chemical model, (B) stress/environmental model, or (C) relationship/interpersonal model. 

Two versions of each of the descriptions incorporating causal models were created: (i) a “simple” description, which was more brief and superficial in its mention of the proposed mechanisms of CBT; and (ii) a “complex” description, which was lengthier and more nuanced in its explanation of the mechanisms of CBT framed within a particular model. Two of the authors were responsible for adapting each of the simple and complex descriptions of CBT within the three causal frameworks described above. The content of each of these descriptions was created based on reliable, evidence-based findings demonstrating links between depression and the proposed mechanisms [44,45,46]. The content was also based on evidenced mechanistic changes in brain functioning and structure, stress appraisal and coping styles, and relationship and interpersonal factors associated with the completion of a course of CBT [47,48]. 

Drafts of the descriptions were presented to three experts with at least five years of experience in their fields. One psychiatrist evaluated the biological descriptions, while one clinical psychologist read the stress descriptions and a second clinical psychologist read the relationship descriptions. These experts provided feedback on the plausibility of the mechanisms proposed as well as the accuracy and readability of the descriptions. Changes suggested by the experts were first implemented in the complex versions of the descriptions. Subsequently, the simple versions were created from the complex descriptions. 

#### 2.2.2. Depression/CBT Beliefs Scale

Nine items categorised into three three-item subscales were developed to assess the extent to which participants agreed with the biology-, relationship-, and stress-oriented explanatory models of depression and the change mechanisms in CBT for depression (e.g., “Cognitive behavioural therapy for depression works to relieve symptoms by changing brain chemistry and structure”). Responses were marked on a Likert scale ranging from 1 (“Completely disagree”) to 7 (“Completely agree”). Three subscales (i.e., the believability of the biology-, relationship-, and stress-based explanatory models) were calculated from the nine items by adding the scores of the items assessing believability within each model. High scores indicated greater endorsements of the respective explanatory model. In the current study, Cronbach’s alpha was 0.75 for the biological model, 0.68 for the relationship model, and 0.71 for the stress model. 

#### 2.2.3. Procedure

After providing their consent, the participants were randomised to one of seven conditions (generic, biological simple, biological complex, relationship simple, relationship complex, stress simple, or stress complex) using Qualtrics’ randomisation feature of random subsets. One of the seven CBT descriptions was randomly displayed to each participant. The participants were subsequently asked to complete the Depression/CBT Beliefs scale. At the end of the survey, they answered questions about their demographic information and were subsequently thanked, debriefed, and compensated USD 2.00 for their participation, which is commensurate with compensation in other crowdsourcing experiments [40]. 

#### 2.2.4. Data Screening and Statistical Analyses

We initially assessed the data for accuracy, missingness, and violations of assumptions. Respondents who failed attention checks and those with more than 20% missing data were excluded from the analyses [49]. In the present dataset, there were no missing data, and all consenting participants passed the included attention-check test. We conducted a MANOVA to test the effect of the type of description provided on depression/CBT beliefs. The treatment description condition was included as the independent variable, and scores on the three-item subscales from the depression/CBT Beliefs scale were included as outcome variables. For findings significant at alpha = 0.05, least significant difference (LSD) post-hoc tests were used to test group differences. 

As part of our preregistered analyses, we also conducted multiple regression analyses to examine whether group assignment predicted depression/CBT believability over and above variance attributed by demographic variables that may correlate with depression and/or CBT perceptions or outcomes [50]. Refer to the Supplementary Information for more details on these statistical analyses. 

#### 2.2.5. Deviations from Pre-Registration

Although the current study matched the pre-registration very closely, some deviations are worth noting. First, we introduced an additional hypothesis predicting that participants receiving complex descriptions will rate the matching explanatory models as more believable than those receiving simple descriptions. Second, our pre-registered sample size was 420 participants, with 60 in each condition; however, our final sample consisted of 425 participants, with only two out of the seven conditions consisting of 60 participants (Table A1). Third, we conducted LSD post-hoc tests to compare group differences instead of the pre-registered independent samples t-tests to control for the family-wise error rate. 

### 2.3. Results 

#### 2.3.1. Effect of Treatment Description on Beliefs

Using Pillai’s trace, a significant multivariate effect of treatment description on depression/CBT beliefs was observed, V = 0.30, F(18, 1254) = 7.75, *p* < 0.001, ηp2 = 0.10. Post-hoc tests revealed a significant difference in the believability of the biological explanatory model of depression/CBT between participants receiving the generic description and participants receiving either of the biological descriptions. Participants who received both the simple and the complex biological treatment descriptions showed greater believability in the biological explanatory model of depression/CBT than those who received the generic treatment description (Table A2). In contrast, there was no difference in the believability of the stress-oriented explanatory model between participants who received the generic treatment description and participants who received either the simple stress-oriented treatment description or the complex stress-oriented treatment description (Table A2). There was also no difference in the believability of the relationship-oriented explanatory model between participants who received the generic treatment description and participants who received the simple relationship-oriented or complex relationship-oriented treatment descriptions (Table A2). Finally, across the three explanatory models, there was no difference in the believability of the matching explanatory models between the participants who received simple treatment descriptions and those who received complex treatment descriptions.

#### 2.3.2. Demographic Predictors and Treatment Descriptions

Zero-order correlations between demographic factors and the believability of CBT mechanisms are presented in Table S1 (Supplementary Information). Hierarchical regressions revealed that treatment description group allocation predicted CBT/depression believability over and above demographic variables for believability of the biological (Table S3) and relationship (Table S4) models but not the stress (Table S5) model. 

Consistent with our predictions, we found that participants randomised to receive a description of CBT conforming to a biological model of depression endorsed heightened believability of the biological causes of depression and mechanisms of CBT. The complexity of the tailoring within the biological model description did not matter; those who received both simple and complex biological explanations believed the biological story of CBT for depression more readily than those who received a generic cognitively oriented description of CBT. Contrary to predictions, neither those randomised to receive descriptions of CBT for depression conforming to the stress-based model nor those randomised to receive a description conforming to the relationship-based model of the disorder endorsed greater believability of their corresponding models or mechanisms to be higher than those who received the generic description. Complexity also did not seem to correspond to differences in the believability of stress-based or relationship-based explanations of depression or mechanisms of CBT. 

Overall, the findings from Study 1 suggest that the believability of CBT mechanisms depends on several contextual and demographic factors. Importantly, once depression is framed within a biological perspective, potential consumers of CBT were less likely to believe that CBT may disrupt these biological mechanisms unless explicitly outlined. 

## 3. Study 2 Method

### 3.1. Participants and Recruitment

An online community sample of 449 participants was recruited using TurkPrime. As in Study 1, participation was restricted to those aged 18 years or older who were fluent in English and resided in an English-speaking country (see Table A3 for participant demographics). 

### 3.2. Materials

#### 3.2.1. CBT Descriptions

For tailored descriptions, the descriptions from Study 1 with the highest total scores on their corresponding items on the Depression/CBT Beliefs scale were used: simple biological, simple stress, and complex relationship. For the generic description, the classic cognitively oriented description from Study 1 was used. 

#### 3.2.2. Modified Treatment Acceptability/Adherence Scale (TAAS)

The TAAS [51] is an eight-item self-report scale used to measure treatment acceptability and the expected ability to adhere to a treatment. In the current study, the statements on the scale were modified by replacing the words “treatment” with “cognitive behavioural therapy” and “fear/anxiety” with “depression” (e.g., “If I participated in Cognitive Behavioural Therapy, I would be able to adhere to its requirements”). Statements were rated on a Likert scale ranging from 1 (“Strongly disagree”) to 8 (“Strongly agree”). The total scores were calculated by adding the responses marked on each item. Higher scores indicated positive perceptions of treatment acceptability. For the modified TAAS, Cronbach’s alpha was 0.85. 

#### 3.2.3. Modified Credibility/Expectancy Questionnaire (CEQ)

The CEQ [17] is a six-item self-report scale used to measure treatment credibility and outcome expectations. In the current study, statements on the scale were modified by replacing the word “therapy” with “cognitive behavioural therapy” (e.g., “At this point, how logical does Cognitive Behavioural Therapy seem to you”). Three items were rated on a 10-point scale from 1 (“Not at all logical”) to 10 (“Very logical”), and the three items were rated on an 11-point scale (from 0% to 100%). The total scores were calculated by adding the responses marked on each item. Higher scores indicated positive perceptions of treatment credibility and expectations. For the modified CEQ, Cronbach’s alpha was 0.91.

### 3.3. Procedure

After providing their consent, the participants were asked to answer the question, “Out of the following list of factors, which do you think is the MOST important cause of depression?”. The list of factors provided contained biology and chemistry, stress in the environment, relationship problems, and other. Respondents indicating “other” causes of depression were ineligible from further participation in the study. The remaining participants were randomised to the tailored condition or the control condition. Participants in the tailored condition received a description of CBT for depression tailored to their endorsement of the most important cause of depression. For example, participants endorsing biological causes of depression at baseline were provided with the simple biological tailored description. Participants in the control condition received the previously described generic, classic description of CBT for depression. After reading the descriptions, participants completed the modified TAAS and CEQ, which were presented in random order. At the end of the survey, participants provided their demographic information and were subsequently thanked, debriefed, and compensated USD 2.5 for their participation.

### 3.4. Data Screening and Analysis Plan

As with Study 1, we scanned the data for accuracy, missingness, and violations of assumptions. There were no missing data. One participant was excluded from the analyses for failing one of the two attention checks. We conducted a MANOVA to test the effect of tailoring on CBT perceptions on the remaining participants (*n* = 449). TAAS and CEQ were entered as outcome variables; the explanatory model endorsed by participants (biological/relationship/stress) and type of description provided (tailored/generic) were entered as independent variables. 

### 3.5. Results 

A total of *n* = 199 (44.3% of the sample included in final analyses) participants selected biology and chemistry as the most important cause of depression, *n* = 208 (46.3%) selected stress in the environment, *n* = 42 (9.4%) selected relationship problems, and *n* = 15 selected other causes. Respondents citing other causes of depression were excluded from further analyses. Of the 199 participants endorsing a biological model, *n* = 103 were randomised to the tailored biological CBT description (while *n* = 96 were randomised to the generic); of the 208 participants endorsing a stress-based model, *n* = 105 were randomised to the tailored stress-based CBT description (while *n* = 103 were randomised to receive the generic description); of the 42 participants endorsing the relationship-based model, *n* = 22 were randomised to the tailored relationship-based CBT description (while 20 were randomised to receive the generic description). 

Using Pillai’s trace, there were no significant main effects of tailoring V = 0.002, F(2, 442) = 0.52, *p* = 0.593, ηp2 = 0.002, or the explanatory model endorsed at baseline by participants, V = 0.02, F(4, 886) = 2.09, *p* = 0.080, ηp2 = 0.01, on the scores of CBT’s acceptability, credibility, and expectancy. The interaction effect of the variables was also non-significant, V = 0.01, F(4, 886) = 0.94, *p* = 0.443, ηp2 = 0.004. Refer to Table A4 for the means and standard deviations of the CEQ and TAAS results. 

Zero-order correlations between demographic factors and perceptions of CBT’s acceptability, credibility, and expectancy are presented in Table S2 (Supplementary Information). Income, being married, and full-time employment correlated significantly with acceptability. There were no statistically significant relationships of the demographic variables with credibility and expectancy. 

Contrary to our hypothesis—that people who received tailored descriptions of CBT would endorse higher acceptability and credibility ratings for the treatment—the results demonstrate that those who received tailored descriptions of CBT did not have significantly different perceptions of CBT than those who received a generic description of the treatment. 

## 4. General Discussion

The current investigation had two goals: first, we sought to develop persuasive psychoeducational materials based on commonly held lay etiological models of depression and examine participants’ belief in CBT’s ability to disrupt mechanisms as described in such models (Study 1). Second, we aimed to examine the effects of providing tailored treatment descriptions on perceptions of CBT’s acceptability and credibility as a treatment for depression (Study 2). In Study 1, participants who received biology-oriented treatment descriptions rated the biological change mechanisms involved in CBT as more believable than those who received a generic cognitively oriented description. The Study 2 hypothesis that participants who received a tailored description would rate CBT more favourably than participants who received a generic description was not supported. 

Evidence points to increasing endorsement of biological models of depression among the general population [52]. Consistent with this, in Study 2, a large minority (44.3%) of participants endorsed a biological model of depression. Lebowitz et al. [53] found that biological explanations of depression symptoms were associated with high prognostic pessimism. Interestingly, this pessimism was reduced significantly once researchers provided participants with an audiovisual intervention regarding the changeability of genetic and neurochemical factors in depression through the use of various evidence-based treatments. Accordingly, if biological explanations of depression are endorsed, patients and other potential consumers of therapy can be readily persuaded that CBT is effective in disrupting even these seemingly indelible mechanisms of the disorder. 

In endorsing the use of psychoeducational material which suggests that biological mechanisms may be disrupted through the effects of a treatment that is otherwise believed to operate through cognitive processes, we also acknowledge the instinctive skepticism surrounding biological bases of CBT. Indeed, not only are the roles of biological processes in treatment mechanisms consistent with the cognitive theory that CBT is built upon [54,55], but evidence also confirms the relationship between CBT and neurobiological changes. Specifically, the evidence suggests that the treatment of depression via CBT is associated with increased functioning in certain areas of the brain (e.g., the hippocampus and dorsal cingulate cortex) and decreased functioning in other areas (e.g., the medial and ventrolateral prefrontal cortex) following treatment [55,56,57]. This is consistent with the messaging in our biological descriptions of CBT. 

The expanding literature, which now demonstrates the meaningful relationships between endorsing biological causes of depression and treatment preference, empowerment in seeking treatment, and prognostic pessimism, is highly suggestive [53,59,60]. Using psychoeducational materials that emphasise the biological change mechanisms involved in CBT, such as the one developed for the purposes of the current investigation, may aid in promoting CBT as a viable treatment option to individuals who endorse such models. Further, a pre-treatment assessment of clients’ explanatory models followed by adapting the treatment rationale in a manner that is congruent with their biological beliefs about depression may help avert some prognostic pessimism stemming from such beliefs. This process may eventually result in improved engagement and treatment outcomes. 

The findings from the current investigation also suggest that individuals already readily believe CBT is able to address relationship and stress mechanisms associated with depression, even in the context of classic cognitively oriented descriptions of the treatment. Importantly, we found that belief in the biological change mechanisms involved in CBT was relatively low across groups. With that, and consistent with Lebowitz et al.’s [53] findings, participants who received biology-oriented descriptions, which explicitly express the malleability of biological mechanisms, rated CBT’s ability to disrupt such mechanisms as more believable than participants who received any other description. 

The believability of the relationship mechanisms of CBT was also predicted by the description allocation beyond demographic variables. Interestingly, those who received a relationship-focused description of CBT specifically did not report higher degrees of believability of the relationship mechanism of treatment. Instead, those who received a complex biological description were less convinced of the proposed relationship mechanisms of CBT than those who received a generic CBT description. This pattern of results is consistent with findings from previous studies demonstrating that participants tend to select psychological interventions (including CBT) as their preferred treatment for depression when endorsing more psychology-oriented causal models [2,31,32]. CBT is intuitively believed to be a psychologically appropriate treatment to disrupt psychological factors, broadly defined. 

Taken together, a consistent pattern of results emerged across the studies (current Study 1 included): (a) people are more reluctant to believe that CBT and/or other psychological interventions operate through biological mechanisms, and promisingly, (b) explicit psychoeducation about why or how CBT (or other interventions) can disrupt biological mechanisms in depression appears effective in improving the believability of the treatment. 

In Study 2, we found no significant difference in perceptions of acceptability, credibility or expectancy between participants receiving a treatment description tailored to match baseline etiological beliefs about depression and those receiving a generic cognitively oriented description. This finding is inconsistent with the results of past work that demonstrated the superiority of tailored health messages over generic ones in promoting a health behaviour change [34]. One interpretation may be that there is indeed no difference or a minimal difference between generic and etiologically tailored descriptions in perceptions of the use of CBT for depression. However, using the elaboration likelihood model [35], Petty et al. [36] suggest that tailored messages are effective tools of persuasion as they prompt individuals to actively engage with personally relevant content. It is therefore more likely that because our descriptions were only tailored in one dimension, the extent of tailoring provided in our descriptions was not sufficient to produce personally relevant content that participants actively engaged with. 

Considering the results of our studies together, tailoring treatment descriptions (to be specifically biologically oriented) appears to improve the believability of the treatment mechanisms but does not improve acceptability and credibility/expectancy beliefs. Although buy-in to the mechanisms of a treatment is likely to have an impact on perceptions of credibility and/or acceptability, beliefs regarding the mechanisms of a treatment are separate from perceptions of its acceptability and credibility. As Khalsa [32] argued, there need not be synchrony between etiological beliefs about depression and treatment preferences. People may endorse biological causes of depression but simultaneously prefer psychotherapy over medication, which could be due to worries about the potential side effects of antidepressants. Similarly, people may believe that a treatment operates through certain mechanisms but may not necessarily rate the treatment positively merely because it aligns with their beliefs about the disorder and/or treatment. 

Our investigation adds to the existing literature in several ways. First, we examined complex and simple versions of the descriptions in Study 1. To our knowledge, this is the only study on beliefs about CBT and depression to compare the effects of relatively superficial descriptions to more elaborate ones. Second, the results of Study 1 also demonstrate that people readily believe in the relationship- and stress-oriented change mechanisms brought about by CBT, even in the context of a generic, classic description of the CBT model; however, generic descriptions of CBT that rely on classic cognitive behavioural mediational hypotheses do little to improve the believability of CBT’s ability to alter biological mechanisms once such mechanisms are invoked or suggested. Finally, to our knowledge, no study has examined the effect of tailoring brief psychoeducational materials on perceptions of the acceptability and credibility of CBT as a treatment for depression. 

The findings from this investigation must be considered in light of its limitations, which pave the way for future research. First, we did not measure the participants’ baseline perceptions of CBT and depression beliefs in Study 1 or baseline treatment perceptions of acceptability or credibility/expectancy in Study 2. As such, we could not ascertain any pre–post changes in beliefs or treatment perceptions of acceptability and credibility/expectancy. Second, in Study 2, the participants were offered a list of only four choices of potential explanatory models at baseline and did not have the choice to endorse multivariate models (e.g., biopsychosocial explanations) as causes for depression. It is possible that a sizable proportion of our sample may have endorsed a multivariate model if provided the option. Third, we relied on crowd-sourced samples. Although such samples confer several advantages (e.g., access to more diverse demographic characteristics and relatively larger samples), criticisms about non-naïveté among participants and the representativeness of the samples remain [40,58]. Our samples were also relatively homogenous in their demographics—the majority of participants identified as white Americans (76% and 78% of the samples in Study 1 and 2, respectively)—undermining the generalisability of our results to racially minoritised and international populations. There is some evidence to suggest that demographic variables, including race, are associated with treatment perceptions and other related constructs (refer to the Supplementary Information for more detail). Thus, a more diverse sample may have improved the generalisability of our findings. 

In light of these limitations, the findings offer directions for further research. Future research may seek to investigate the effect of tailoring psychoeducational materials on multiple dimensions simultaneously. These dimensions may be based on an assessment of prior experience with CBT and/or other psychotherapies, individual differences (e.g., psychological mindedness and self-efficacy), perceived barriers to CBT (e.g., stigma and misconceptions), and demographic characteristics (e.g., educational attainment), to name a few. Additionally, considering the popularity of biological models of depression in the general population, ways of improving the current biology-oriented descriptions or the efficacy of alternate psychoeducational interventions may be explored. It may also be worth using longitudinal designs to examine whether effects on beliefs persist at follow-up assessments and whether they materialise into behavioural change when seeking treatment. Lastly, distributional channels may be compared through ecologically valid and/or naturalistic formats such as websites [24] or brochures in clinics. 

## 5. Conclusions

In this two-study investigation, we examined the interplay between lay etiological models of depression and treatment perceptions following the administration of brief psychoeducational interventions. Our results demonstrated that across groups, participants readily believed in stress- and relationship-oriented change mechanisms of CBT for depression despite the provision of a classic cognitively oriented treatment description. Our results also revealed that beliefs in CBT’s ability to disrupt biological mechanisms of depression are generally low; however, they improve with the presentation of a biology-oriented treatment description. Further, etiologically tailored treatment descriptions did not result in more favourable perceptions of CBT as an acceptable and credible treatment for depression. Our findings elucidate the nature of beliefs about CBT as a treatment for depression, specifically in the context of lay biological explanatory models of the disorder.

## Data Availability

The datasets corresponding to both studies are publicly available on the Open Science Framework (osf.io) [https://doi.org/10.17605/OSF.IO/UTN2Z]. Accessed on 8 July 2023.

## Supplementary Materials

The following supporting information can be downloaded at: https://www.mdpi.com/article/10.3390/ijerph20146330/s1; the supplementary materials include the CBT descriptions and analyses pertaining to demographic predictors. Table S1: Correlations Between Demographic Variables and CBT Believability; Table S2: Correlations Between Demographic Variables and scores on TAAS and CEQ; Table S3: Summary of Hierarchical Regression for Biology Believability with Standardised Coefficients; Table S4: Summary of Hierarchical Regression for Relationship Believability with Standardised Coefficients; Table S5: Summary of Hierarchical Regression for Stress Believability with Standardised Coefficients.

## Appendix A

**Table A1:** Study 1 sample demographics.

	Total (*n* = 425)	Generic (*n* = 59)	Simple			Complex		
			Biology (*n* = 58)	Stress (*n* = 59)	Relationship (*n* = 66)	Biology (*n* = 60)	Stress (*n* = 60)	Relationship (*n* = 63)
Age: *M(SD)*	35.8 (11.2)	36.5 (11.5)	36.9 (12.0)	36.1 (11.0)	34.3 (11.0)	35.3 (11.9)	35.4 (9.7)	36.7 (11.4)
Race/Ethnicity ^a^: *n* (%)								
White	323 (76)	51 (86.4)	45 (77.6)	45 (76.3)	46 (69.7)	44 (73.3)	51 (85)	42 (66.7)
Black	56 (13.2)	5 (8.5)	5 (8.6)	8 (13.6)	7 (10.6)	8 (13.3)	7 (11.7)	17 (27.0)
Asian	35 (8.2)	6 (10.2)	6 (10.3)	6 (10.2)	8 (12.1)	4 (6.7)	3 (5.0)	5 (7.9)
Hispanic/Latino	29 (6.8)	1 (1.7)	5 (8.6)	4 (6.8)	5 (7.6)	7 (11.7)	4 (6.7)	3 (4.8)
Indigenous	4 (0.9)	0 (0)	1 (1.7)	1 (1.7)	0 (0)	0 (0)	1 (1.7)	1 (1.6)
Middle Eastern/North African	3 (0.7)	0 (0)	1 (1.7)	1 (1.7)	1 (1.5)	0 (0)	0 (0)	0 (0)
Other	3 (0.7)	0 (0)	0 (0)	0 (0)	2 (3.0)	0 (0)	1 (1.7)	
Gender								
Male	226 (53.2)	28 (47.5)	30 (51.7)	27 (45.8)	40 (60.6)	32 (53.3)	31 (51.7)	38 (60.3)
Female	198 (46.6)	31 (52.5)	28 (48.3)	32 (54.2)	25 (37.9)	28 (46.7)	29 (48.3)	25 (39.7)
Non-binary	1 (0.2)	0 (0)	0 (0)	0 (0)	1 (1.5)	0 (0)	0 (0)	0 (0)
Country								
Canada	2 (0.5)	0 (0)	0 (0)	1 (1.7)	0 (0)	1 (1.7)	0 (0)	0 (0)
USA	423 (99.5)	59 (100)	58 (100)	58 (98.3)	66 (100)	59 (98.3)	60 (100)	63 (100)
Education								
High school or below	133 (31.3)	15 (25.4)	15 (25.9)	14 (23.7)	24 (36.4)	27 (45.0)	18 (30.0)	20 (31.7)
College	230 (54.1)	37 (62.7)	35 (60.3)	37 (62.7)	33 (50)	25 (41.7)	33 (55.0)	30 (47.6)
Postgraduate	62 (14.6)	7 (11.9)	8(13.8)	8 (13.6)	9 (13.6)	8 (13.3)	9 (15.0)	13 (20.6)
Marital Status								
Single	167 (39.3)	25 (42.4)	28 (48.3)	11 (18.6)	27 (40.9)	28 (46.7)	18 (30.0)	30 (47.6)
Dating	35 (8.2)	7 (11.9)	2 (3.4)	8 (13.6)	6 (9.1)	2 (3.3)	6 (10.0)	4 (6.3)
Married/cohabitating	199 (46.8)	27 (45.8)	22 (37.9)	39 (66.1)	27 (40.9)	26 (43.3)	30 (50.0)	28 (44.4)
Divorced/separated	23 (5.4)	0 (0)	5 (8.6)	1 (1.7)	6 (9.1)	4 (6.7)	6 (10)	1 (1.6)
Widowed	1 (0.2)	0 (0)	1 (1.7)	0 (0)	0 (0)	0 (0)	0 (0)	0 (0)
Employment								
Full-time	313 (73.6)	45 (76.3)	43 (74.1)	46 (78.0)	44 (66.7)	45 (75.0)	45 (75)	45 (71.4)
Part-time	51 (12)	7 (11.9)	6 (10.3)	6 (10.2)	11 (16.7)	6 (10.0)	8 (13.3)	7 (11.1)
Unemployed	22 (5.2)	2 (3.4)	4 (6.9)	6 (10.2)	2 (3.0)	2 (3.3)	2 (3.3)	4 (6.3)
Not looking for work	21 (4.9)	3 (5.1)	1 (1.7)	1 (1.7)	0 (0)	3 (5.0)	2 (2.2)	4 (6.3)
Never employed	6 (1.4)	1 (1.7)	0 (0)	0 (0)	1 (1.5)	2 (3.3)	1 (1.7)	1 (1.6)
Retired	12 (2.8)	1 (1.7)	4 (6.9)	0 (0)	1 (1.5)	2 (3.3)	2 (3.3)	2 (3.2)
Previously familiar with the term “CBT”	203 (47.8)	27 (45.8)	28 (48.3)	30 (50.8)	32 (48.5)	30 (50)	24 (40)	32 (50.8)
Previously treated for depression	128 (30.1)	18 (30.5)	15 (25.9)	16 (27.1)	23 (34.8)	20 (33.3)	16 (26.7)	20 (31.7)
Previoussly treated with CBT for depression ^b^	52 (40.6)	4 (22.2)	6 (40)	8 (50)	11 (47.8)	11 (55)	4 (25)	8 (40)

Note: ^a^ Participants could select multiple options; ^b^ percentage derived from participants previously treated for depression.

**Table A2:** Means, standard deviations, and *p*-values of differences in believability of explanatory models.

Biological Model									
Treatment Description	M	SD	1	2	3	4	5	6	7
Generic	12.29	4.72		<0.001	<0.001	0.096	0.341	0.005	0.430
Biological, simple	15.93	3.41			0.826	<0.001	<0.001	<0.001	<0.001
Biological, complex	15.77	3.56				<0.001	<0.001	<0.001	<0.001
Relationship, simple	11.08	4.17					0.475	0.214	0.389
Relationship, complex	11.59	4.49						0.055	0.878
Stress, simple	10.17	4.20							0.041
Stress, complex	11.70	3.67							
Stress-oriented Model									
Treatment description	M	SD	1	2	3	4	5	6	7
Generic	17.81	2.26		0.401	0.059	0.039	0.237	0.355	0.844
Biological, simple	17.40	2.69			0.300	0.232	0.746	0.079	0.517
Biological, complex	16.88	2.36				0.892	0.464	0.005	0.090
Relationship, simple	16.82	3.32					0.375	0.003	0.061
Relationship, complex	17.24	2.39						0.034	0.323
Stress, simple	18.27	2.68							0.260
Stress, complex	17.72	2.86							
Relationship-oriented Model									
Treatment description	M	SD	1	2	3	4	5	6	7
Generic	16.88	2.92		0.712	0.031	0.469	0.247	0.763	0.086
Biological, simple	16.67	2.75			0.075	0.733	0.127	0.944	0.180
Biological, complex	15.67	3.16				0.134	0.001	0.063	0.654
Relationship, simple	16.48	3.51					0.054	0.679	0.298
Relationship, complex	17.52	2.55						0.143	0.004
Stress, simple	16.71	3.25							0.157
Stress, complex	15.92	3.11							

**Table A3:** Study 2 sample demographics.

	Total (*n* = 449)	Generic (*n* = 219)	Tailored		
			Biology (*n* = 103)	Stress (*n* = 105)	Relationship (*n* = 22)
Age: *M(SD)*	38.1 (12)	37.79 (12.3)	40.0 (12.2)	36.9 (11.3)	37.5 (10.1)
Race/Ethnicity ^a^: *n* (%)					
White	361 (80.4)	174 (79.5)	93 (90.3)	80 (76.2)	13 (59.1)
Black	40 (8.9)	24 (11.0)	4 (3.9)	10 (9.5)	2 (9.1)
Asian	44 (9.8)	16 (7.3)	7 (6.8)	14 (13.3)	6 (27.3)
Hispanic/Latino	19 (4.2)	11 (5.0)	1 (1.0)	7 (6.7)	0 (0)
Indigenous	4 (0.9)	1 (0.5)	1 (1.0)	0 (0)	1 (4.5)
Middle Eastern/North African	2 (0.4)	0 (0)	0 (0)	1 (1.0)	1 (4.5)
Other	1 (0.2)	1 (0.5)	2 (1.9)	2 (1.9)	0 (0)
Gender					
Male	216 (48.1)	107 (48.9)	39 (37.9)	62 (59.0)	8 (36.4)
Female	232 (51.7)	111 (50.7)	64 (62.1)	43 (41.0)	14 (63.6)
Non-binary	1 (0.2)	1 (0.5)	0 (0)	0 (0)	0 (0)
Country					
Canada	10 (2.2)	4 (1.8)	2 (1.9)	4 (3.8)	0 (0)
UK	2 (0.4)	2 (0.9)	0 (0)	0 (0)	0 (0)
USA	437 (97.3)	213 (97.3)	101 (98.1)	101 (96.2)	22 (100)
Education					
High school or below	125 (27.8)	15 (25.4)	26 (25.2)	27 (25.7)	8 (36.4)
College	260 (57.9)	37 (62.7)	61 (59.2)	59 (56.2)	10 (45.5)
Postgraduate	64 (14.3)	7 (11.9)	16 (15.5)	19 (18.1)	4 (18.2)
Marital Status					
Single	165 (36.7)	88 (40.2)	32 (31.1)	41 (39.0)	4 (18.2)
Dating	43 (9.6)	27 (12.3)	6 (5.8)	7 (6.7)	3 (13.6)
Married/cohabitating	199 (44.3)	83 (37.9)	53 (51.5)	48 (45.7)	15 (68.2)
Divorced/separated	38 (8.5)	19 (8.7)	11 (10.7)	8 (7.6)	0 (0)
Widowed	4 (0.9)	2 (0.9)	1 (1.0)	1 (1.0)	0 (0)
Employment					
Full-time	287 (63.9)	141 (64.4)	63 (61.2)	74 (70.5)	9 (40.9)
Part-time	79 (17.6)	36 (16.4)	23 (22.3)	14 (13.3)	6 (27.3)
Unemployed	26 (5.8)	13 (5.9)	3 (2.9)	9 (8.6)	1 (4.5)
Not looking for work	35 (7.8)	19 (8.7)	7 (6.8)	4 (3.8)	5 (22.7)
Never employed	1 (0.2)	0 (0)	0 (0)	1 (1.0)	0 (0)
Retired	21 (4.7)	10 (4.6)	7 (6.8)	3 (2.9)	1 (4.5)
Previously familiar with the term ‘CBT’	204 (45.4)	102 (46.6)	46 (44.7)	48 (45.7)	8 (36.4)
Previously treated for depression	167 (37.2)	82 (37.4)	39 (37.9)	39 (37.1)	7 (31.8)
Previously treated with CBT for depression ^b^	52 (31.1)	23 (28.0)	12 (30.8)	13 (33.3)	4 (57.1)

Note: ^a^ Participants could select multiple options; ^b^ percentage derived from participants previously treated for depression.

**Table A4:** Means and standard deviations of scores on the CEQ and TAAS.

	CEQ				TAAS			
	Tailored		Control		Tailored		Control	
	*M*	*SD*	*M*	*SD*	*M*	*SD*	*M*	*SD*
Biochemical	36.30	9.24	35.90	10.70	36.60	8.22	36.48	9.10
Stress/Environmental	38.87	8.02	37.94	9.14	38.23	7.39	38.24	7.96
Relationship/Interpersonal	36.68	10.72	41.45	9.64	37.49	6.63	39.30	7.67
