# Supplementary material for: Framing Effects of Cognitive Behavioural Therapy for Depression on Perceptions of Believability, Acceptability, and Credibility
Source: Int J Environ Res Public Health. 2023 Jul 9;20(14):6330. doi: 10.3390/ijerph20146330 (PMC10379820; doi:10.3390/ijerph20146330)
Supplement: Supplementary file 1 [file ijerph-20-06330-s001.zip › ijerph-2264810-supplementary.pdf]

Supplementary Information

CBT Descriptions

Description 1: Biology (Complex Version)

Cognitive behavior therapy (CBT) is a type of therapy that helps reduce symptoms of depression. CBT focuses on helping patients understand how thoughts and behaviors impact brain structure and chemistry, which directly affects emotions like depression. The human brain is composed of specific structures that directly influence our mood. Brain structures communicate with one another other using different chemicals. Depression is linked with abnormal brain structures and chemical imbalances in the brain. Over the course of treatment, CBT can increase the function of specific brain structures and help regulate chemical imbalances in the brain by correcting the negative thoughts and behaviors of depression. The goal of CBT is to change thoughts and behaviors to help improve brain structure and chemistry, which then reduces symptoms of depression. CBT often happens in a therapist's office. In order to give you a sense of CBT, we would like to provide you with specific information about how CBT for depression is done. CBT for depression typically consists of 12 to 20 face-to-face sessions, wherein a depressed patient meets on a weekly basis with a qualified cognitive-behavioral therapist. In the first few therapy sessions, patients are provided facts about depression and on common depression related thoughts and behaviors. In later sessions, the therapist helps the patient learn new ways to cope with depression. The therapist helps the patient identify and change negative thoughts that were untrue or unhelpful (e.g., "I will always be alone"). The patient and therapist also work together to create a list of activities that should be accomplished during the course of treatment. The activities are either important for day-to-day functioning (e.g., paying the electric bill) or pleasurable for the patient (e.g., going to a party with friends). The patient then gradually works on accomplishing these tasks and goals, beginning with something that was small (e.g., bathing, cooking) and moving to more difficult tasks (e.g., scheduling "date-nights"). Treatment completes with patients developing a plan for how they would cope if symptoms of depression reappeared. Skills are first practiced in therapy sessions with the therapist present. Patients are assigned homework exercises at the end of each session. The homework exercises included tracking symptoms, thoughts, and behaviors related to depression, as well as completing important and pleasurable tasks outside of the therapist's office. The amount of time needed for homework varies for each patient, but typically requires at least some time each day.

Advantages of CBT:

1. According to scientific studies, most patients (55-65%) with depression who undergo CBT see significant reductions in their depression symptoms, in comparison to patients who are not offered any treatment.
2. There is scientific evidence to suggest that the skills gained during a course of CBT for depression (e.g., identifying and challenging negative thoughts and behaviors) directly improves brain structures and chemical imbalances in the brain.
3. The therapist can easily adapt information to the needs of the patient.
4. CBT for depression has been successfully delivered remotely via technology (i.e., online or over the phone).
5. CBT for depression has no biological side-effects.

Disadvantages of CBT:

1. A large minority (35-45%) of patients who undergo CBT for depression do not experience significant reductions in their symptoms
2. After one year of ending treatment, about a quarter of the patients (25%) who first respond to CBT experience a return of some depression symptoms.
3. CBT requires time and effort in order to reduce depression.
4. The success of CBT for depression depends to a large degree on the delivery techniques of the therapist.
5. To date, there is little scientific consensus as to why, how, or for whom CBT may work.

Description 2: Biology (Simple Version)

Cognitive behavior therapy (CBT) is a type of therapy that helps reduce symptoms of depression. CBT focuses on helping patients understand how thoughts and behaviors impact brain structure and chemistry, which directly affects emotions like depression. Patients with depression may have thoughts that they are not loved or wanted, or that they are worthless. In terms of behaviors, patients may withdraw from relationships, or neglect important tasks like their health and hygiene. Patients may also stop doing pleasurable or enjoyable activities. These thoughts and behaviors are often linked to abnormal brain structure and chemistry, which CBT can correct over the course of treatment. The goal of CBT is to change thoughts and behaviors to help improve brain structure and chemistry, which then reduces symptoms of depression. CBT often happens in a therapist's office. In order to give you a sense of CBT, we would like to provide you with specific information about how CBT for depression is done. CBT for depression typically consists of 12 to 20 face-to-face sessions, wherein a depressed patient meets on a weekly basis with a qualified cognitive-behavioral therapist. In the first few therapy sessions, patients are provided facts about depression and on common depression related thoughts and behaviors. In later sessions, the therapist helps the patient learn new ways to cope with depression. The therapist helps the patient identify and change negative thoughts that were untrue or unhelpful (e.g., "I will always be alone"). The patient and therapist also work together to create a list of activities that should be accomplished during the course of treatment. The activities are either important for day-to-day functioning (e.g., paying the electric bill) or pleasurable for the patient (e.g., going to a party with friends). The patient then gradually works on accomplishing these tasks and goals, beginning with something that was small (e.g., bathing, cooking) and moving to more difficult tasks (e.g., scheduling "date-nights"). Treatment completes with patients developing a plan for how they would cope if symptoms of depression reappeared. Skills are first practiced in therapy sessions with the therapist present. Patients are assigned homework exercises at the end of each session. The homework exercises included tracking symptoms, thoughts, and behaviors related to depression, as well as completing important and pleasurable tasks outside of the therapist's office. The amount of time needed for homework varies for each patient, but typically requires at least some time each day.

Advantages of CBT:

1. According to scientific studies, most patients (55-65%) with depression who undergo CBT see significant reductions in their depression symptoms, in comparison to patients who are not offered any treatment.
2. There is scientific evidence to suggest that the skills gained during a course of CBT for depression (e.g., identifying and challenging negative thoughts and behaviors) directly improves brain structures and chemical imbalances in the brain
3. The therapist can easily adapt information to the needs of the patient.
4. CBT for depression has been successfully delivered remotely via technology (i.e., online or over the phone).
5. CBT for depression has no biological side-effects.

Disadvantages of CBT:

1. A large minority (35-45%) of patients who undergo CBT for depression do not experience significant reductions in their symptoms
2. After one year of ending treatment, about a quarter of the patients (25%) who first respond to CBT experience a return of some depression symptoms.
3. CBT requires time and effort in order to reduce depression.
4. The success of CBT for depression depends to a large degree on the delivery techniques of the therapist.
5. To date, there is little scientific consensus as to why, how, or for whom CBT may work.

Description 3: Stress (Simple Version)

Cognitive behavior therapy (CBT) is a type of therapy that helps reduce symptoms of depression. CBT focuses on helping patients understand how thoughts and behaviors about stressful situations can affect emotions like depression. People experience different life stressors, including problems with finances, relationships, employment, education, and health. How people respond to stressful situations can directly influence their mood. People can control their stress level by coping with stressful situations. Patients with depression frequently use negative coping strategies to handle life stressors, which can create even more stress in their lives. CBT can help improve positive coping strategies when handling stressful situations. The goal of CBT is to change thoughts and behaviors that directly impact one’s stress level to help reduce symptoms of depression. CBT often happens in a therapist’s office. In order to give you a sense of CBT, we would like to provide you with specific information about how CBT for depression is done. CBT for depression typically consists of 12 to 20 face-to-face sessions, wherein a depressed patient meets on a weekly basis with a qualified cognitive-behavioral therapist. In the first few therapy sessions, patients are provided facts about depression and on common depression related thoughts and behaviors. In later sessions, the therapist helps the patient learn new ways to cope with depression. The therapist helps the patient identify and change negative thoughts that were untrue or unhelpful (e.g., “I will always be alone”). The patient and therapist also work together to create a list of social activities that should be accomplished during the course of treatment. The activities are either important for day-to-day functioning (e.g., paying the electric bill) or pleasurable for the patient (e.g., going to a party with friends). The patient then gradually works on accomplishing these tasks and goals, beginning with something that was small (e.g., bathing, cooking) and moving to more difficult tasks (e.g., scheduling “date-nights”). Treatment

completes with patients developing a plan for how they would cope if symptoms of depression reappeared. Skills are first practiced in therapy sessions with the therapist present. Patients are assigned homework exercises at the end of each session. The homework exercises included tracking symptoms, thoughts, and behaviors related to depression, as well as completing important and pleasurable tasks outside of the therapist's office. The amount of time needed for homework varies for each patient, but typically requires at least some time each day.

Advantages of CBT:

1. According to several scientific studies, most patients (55-65%) of depression who undergo CBT see significant reductions in their depression symptoms, in comparison to patients who are not offered any treatment.
2. There is scientific evidence to suggest that patients of CBT for depression often report more positive coping strategies in response to stressful events, better communication skills with family, improved relationship satisfaction, and improved productivity at work.
3. The therapist can easily adapt information to the needs of the patient.
4. CBT for depression has been successfully delivered remotely via technology (i.e., online or over the phone).
5. CBT for depression has no biological side-effects.

Disadvantages of CBT:

1. A large minority (35-45%) of patients who undergo CBT for depression do not experience significant reductions in their symptoms
2. After one year of ending treatment, about a quarter of the patients (25%) who first respond to CBT experience a return of some depression symptoms.
3. CBT requires time and effort in order to reduce depression.
4. The success of CBT for depression depends to a large degree on the delivery techniques of the therapist.
5. To date, there is little scientific consensus as to why, how, or for whom CBT may work.

Description 4: Stress (Complex Version)

Cognitive behavior therapy (CBT) is a type of therapy that helps reduce symptoms of depression. CBT focuses on helping people understand how thoughts and behaviors about stressful situations can affect emotions like depression. People can control their stress level by coping with stressful situations. People with depression frequently use negative coping strategies to handle stressful situations, which include repeatedly thinking about a stressor without problem-solving, procrastination, emotional eating, employment absenteeism, anger, and substance use. As a result, people with depression may create even more stress in their lives. CBT can help improve positive coping strategies when handling stressful situations. The goal of CBT is to change thoughts and behaviors that directly impact one's stress level to help reduce symptoms of depression. CBT often happens in a therapist's office. In order to give you a sense of CBT, we would like to provide you with specific information about how CBT for depression is done. CBT

for depression typically consists of 12 to 20 face-to-face sessions, wherein a depressed patient meets on a weekly basis with a qualified cognitive-behavioral therapist. In the first few therapy sessions, patients are provided facts about depression and on common depression related thoughts and behaviors. In later sessions, the therapist helps the patient learn new ways to cope with depression. The therapist helps the patient identify and change negative thoughts that were untrue or unhelpful (e.g., "I will always be alone"). The patient and therapist also work together to create a list of social activities that should be accomplished during the course of treatment. The activities are either important for day-to-day functioning (e.g., paying the electric bill) or pleasurable for the patient (e.g., going to a party with friends). The patient then gradually works on accomplishing these tasks and goals, beginning with something that was small (e.g., bathing, cooking) and moving to more difficult tasks (e.g., scheduling "date-nights"). Treatment completes with patients developing a plan for how they would cope if symptoms of depression reappeared. Skills are first practiced in therapy sessions with the therapist present. Patients are assigned homework exercises at the end of each session. The homework exercises included tracking symptoms, thoughts, and behaviors related to depression, as well as completing important and pleasurable tasks outside of the therapist's office. The amount of time needed for homework varies for each patient, but typically requires at least some time each day.

Advantages of CBT:

1. According to several scientific studies, most patients (55-65%) of depression who undergo CBT see significant reductions in their depression symptoms, in comparison to patients who are not offered any treatment.
2. There is scientific evidence to suggest that patients of CBT for depression often report more positive coping strategies in response to stressful events, better communication skills with family, improved relationship satisfaction, and improved productivity at work.
3. The therapist can easily adapt information to the needs of the patient.
4. CBT for depression has been successfully delivered remotely via technology (i.e., online or over the phone).
5. CBT for depression has no biological side-effects.

Disadvantages of CBT:

1. A large minority (35-45%) of patients who undergo CBT for depression do not experience significant reductions in their symptoms
2. After one year of ending treatment, about a quarter of the patients (25%) who first respond to CBT experience a return of some depression symptoms.
3. CBT requires time and effort in order to reduce depression.
4. The success of CBT for depression depends to a large degree on the delivery techniques of the therapist.
5. To date, there is little scientific consensus as to why, how, or for whom CBT may work.

Description 5: Social (Simple Version)

Cognitive behavior therapy (CBT) is a type of therapy that helps reduce symptoms of depression. CBT focuses on helping patients understand how thoughts and behaviors about relationships can affect emotions like depression. Patients with depression may have thoughts that they are not loved or wanted by those they care about, or that they do not belong with other people. In terms of behaviors, patients with depression may withdraw from relationships or stop doing enjoyable activities, such as socializing with friends or going on dates. The goal of CBT is to change thoughts and behaviors that directly impact social relationships in order to reduce symptoms of depression. CBT often happens in a therapist's office. In order to give you a sense of CBT, we would like to provide you with specific information about how CBT for depression is done. CBT for depression typically consists of 12 to 20 face-to-face sessions, wherein a depressed patient meets on a weekly basis with a qualified cognitive-behavioral therapist. In the first few therapy sessions, patients are provided facts about depression and on common depression related thoughts and behaviors. In later sessions, the therapist helps the patient learn new ways to cope with depression. The therapist helps the patient identify and change negative thoughts that were untrue or unhelpful (e.g., "I will always be alone"). The patient and therapist also work together to create a list of social activities that should be accomplished during the course of treatment. The activities are either important for day-to-day functioning (e.g., paying the electric bill) or pleasurable for the patient (e.g., going to a party with friends). The patient then gradually works on accomplishing these tasks and goals, beginning with something that was small (e.g., bathing, cooking) and moving to more difficult tasks (e.g., scheduling "date-nights"). Treatment completes with patients developing a plan for how they would cope if symptoms of depression reappeared. Skills are first practiced in therapy sessions with the therapist present. Patients are assigned homework exercises at the end of each session. The homework exercises included tracking symptoms, thoughts, and behaviors related to depression, as well as completing important and pleasurable tasks outside of the therapist's office. The amount of time needed for homework varies for each patient, but typically requires at least some time each day.

Advantages of CBT:

1. According to several scientific studies, most patients (55-65%) with depression who undergo CBT see significant reductions in their depression symptoms, in comparison to patients who are not offered any treatment.
2. There is scientific evidence to suggest that patients treated with CBT for depression often report higher relationship satisfaction, better communication skills, and increased social activities over the course of treatment.
3. The therapist can easily adapt information to the needs of the patient.
4. CBT for depression has been successfully delivered remotely via technology (i.e., online or over the phone).
5. CBT for depression has no biological side-effects.

Disadvantages of CBT:

1. A large minority (35-45%) of patients who undergo CBT for depression do not experience significant reductions in their symptoms
2. After one year of ending treatment, about a quarter of the patients (25%) who first respond to CBT experience a return of some depression symptoms.

3. CBT requires time and effort in order to reduce depression.
4. The success of CBT for depression depends to a large degree on the delivery techniques of the therapist.
5. To date, there is little scientific consensus as to why, how, or for whom CBT may work.

Description 6: Social (Complex Version)

Cognitive behavior therapy (CBT) is a type of therapy that helps reduce symptoms of depression. CBT focuses on helping patients understand how thoughts and behaviors about relationships can affect emotions like depression. Humans are driven to connect with others. However, patients with depression frequently isolate themselves from others, which can lead to increased loneliness and a lack of belonginess with others, poor self-esteem, jealousy, and social anxiety. Patients with depression may also experience relationship dissatisfaction with intimate partners as well as communication problems, which include expressing negative emotions towards other people, frequent interruptions, critical comments, defensiveness, and poor nonverbal communication skills. The goal of CBT is to change thoughts and behaviors that directly impact social relationships in order to reduce symptoms of depression. CBT often happens in a therapist's office. In order to give you a sense of CBT, we would like to provide you with specific information about how CBT for depression is done. CBT for depression typically consists of 12 to 20 face-to-face sessions, wherein a depressed patient meets on a weekly basis with a qualified cognitive-behavioral therapist. In the first few therapy sessions, patients are provided facts about depression and on common depression related thoughts and behaviors. In later sessions, the therapist helps the patient learn new ways to cope with depression. The therapist helps the patient identify and change negative thoughts that were untrue or unhelpful (e.g., "I will always be alone"). The patient and therapist also work together to create a list of social activities that should be accomplished during the course of treatment. The activities are either important for day-to-day functioning (e.g., paying the electric bill) or pleasurable for the patient (e.g., going to a party with friends). The patient then gradually works on accomplishing these tasks and goals, beginning with something that was small (e.g., bathing, cooking) and moving to more difficult tasks (e.g., scheduling "date-nights"). Treatment completes with patients developing a plan for how they would cope if symptoms of depression reappeared. Skills are first practiced in therapy sessions with the therapist present. Patients are assigned homework exercises at the end of each session. The homework exercises included tracking symptoms, thoughts, and behaviors related to depression, as well as completing important and pleasurable tasks outside of the therapist's office. The amount of time needed for homework varies for each patient, but typically requires at least some time each day.

Advantages of CBT:

1. According to several scientific studies, most patients (55-65%) with depression who undergo CBT see significant reductions in their depression symptoms, in comparison to patients who are not offered any treatment.

2. There is scientific evidence to suggest that patients treated with CBT for depression often report higher relationship satisfaction, better communication skills, and increased social activities over the course of treatment.
3. The therapist can easily adapt information to the needs of the patient.
4. CBT for depression has been successfully delivered remotely via technology (i.e., online or over the phone).
5. CBT for depression has no biological side-effects.

Disadvantages of CBT:

1. A large minority (35-45%) of patients who undergo CBT for depression do not experience significant reductions in their symptoms
2. After one year of ending treatment, about a quarter of the patients (25%) who first respond to CBT experience a return of some depression symptoms.
3. CBT requires time and effort in order to reduce depression.
4. The success of CBT for depression depends to a large degree on the delivery techniques of the therapist.
5. To date, there is little scientific consensus as to why, how, or for whom CBT may work.

Description 7: Generic

Cognitive behavior therapy (CBT) is a type of therapy that research evidence indicates reduces symptoms of depression. CBT focuses on helping patients understand how certain thoughts and actions affect emotions, like intense sadness or depression. Patients with depression may have thoughts that they are not loved or wanted, or that they are worthless. In terms of behaviors, patients may withdraw from the world around them, or neglect important tasks like their health and hygiene. Patients may also stop doing pleasurable or enjoyable activities. The goal of CBT is to change thoughts and behaviors in ways that will help reduce depression. CBT often happens in a therapist's office. In order to give you a sense of CBT, we would like to provide you with specific information about how CBT for depression is done. CBT for depression typically consists of 12 to 20 face-to-face sessions, wherein a depressed patient meets on a weekly basis with a qualified cognitive-behavioral therapist. In the first few therapy sessions, patients are provided facts about depression and on common depression related thoughts and behaviors. In later sessions, the therapist helps the patient learn new ways to cope with depression. The therapist helps the patient identify and change negative thoughts that were untrue or unhelpful (e.g., "I am worthless"). The patient and therapist also work together to create a list of activities that should be accomplished during the course of treatment. The activities are either important for day-to-day functioning (e.g., paying the electric bill) or pleasurable for the patient (e.g., going for a walk, cycling). The patient then gradually works on accomplishing these tasks and goals, beginning with something that was small (e.g., bathing, cooking) and moving to more difficult tasks (e.g., being more assertive at work). Treatment completes with patients developing a plan for how they would cope if symptoms of depression reappeared. Skills are first practiced in therapy sessions with the therapist present. Patients are assigned homework exercises at the end

of each session. The homework exercises included tracking symptoms, thoughts, and behaviors related to depression, as well as completing important and pleasurable tasks outside of the therapist's office. The amount of time needed for homework varies for each patient, but typically requires at least some time each day.

Advantages of CBT:

1. According to several scientific studies, most patients (55-65%) of depression who undergo CBT see significant reductions in their depression symptoms, in comparison to patients who are not offered any treatment.
2. The skills gained during a course of CBT for depression (e.g., identifying and challenging negative thoughts) may have positive impact on a lot of different aspects of patients' lives.
3. The therapist can easily adapt information to the needs of the patient.
4. CBT for depression has been successfully delivered remotely via technology (i.e., online or over the phone).
5. CBT for depression has no physiological side-effects.

Disadvantages of CBT:

1. A large minority (35-45%) of patients who undergo CBT for depression do not experience significant reductions in their symptoms
2. After one year of ending treatment, about a quarter of the patients (25%) who first respond to CBT experience a return of some depression symptoms.
3. CBT requires time and effort in order to reduce depression.
4. The success of CBT for depression depends to a large degree on the delivery techniques of the therapist.
5. To date, there is little scientific consensus as to why, how, or for whom CBT may work.

Demographic Correlates

The causes people attribute to mental illnesses are related to specific demographic factors. For example, beliefs about causes for mental illnesses seem to be at least partially mediated by culture (Choudhry et al., 2016). In line with this finding, researchers have found race and/or ethnicity factors predict beliefs regarding causes for depression. For example, White individuals are more likely to endorse biological causes for depression compared to minoritized individuals (Khalsa et al., 2011). However, no significant associations between race and endorsement of etiological models of depression were found in other studies (O'Connor & Vaughan, 2021). Moreover, although in some studies researchers have found gender differences in the endorsement of the causes of depression, the nature of these differences has not been consistently replicated across studies (Hansson et al., 2010; Khalsa et al., 2011; Schweizer et al., 2010). Likewise, in some studies, researchers have found that older age was associated with lower endorsement of social (i.e., depression is caused by social isolation and/or relationship problems) and stress (i.e., depression is caused by stressful events) models of depression but this also was not replicated in other studies (Hansson et al., 2010; O'Connor & Vaughan, 2021). Accordingly, and given such inconsistent and contradictor findings, high powered studies examining the nature of the relationships between demographic variables and treatment perception factors (believability, acceptability, credibility) are warranted.

Method

Statistical Analyses

Pearson-product moment correlation was conducted for age. Point-biserial correlation was conducted for each level of the categorical variables (i.e., gender, marital status, and

employment). Finally, Spearman's rank correlation was conducted for ordinal variables (i.e., income and education levels).

Three hierarchical regressions were conducted with demographic variables of age, income, education, gender, marital status and employment status entered in block 1, and treatment description condition entered in block 2. Outcome variables were believability of biology, relationship and stress mechanisms of CBT/depression respectively. Since only one participant identified as non-binary and one as widowed, these participants were excluded from the regression analyses.

Results

Demographic correlates of CBT believability, acceptability and credibility are presented in Tables S1 and S2.

Hierarchical regressions revealed that treatment description group allocation predicted CBT/depression believability over and above demographic variables for believability of the biological (Table S3) and relationship (Table S4) models but not the stress model. Believability of the stress-oriented model (Table S5) was significantly predicted by the demographic variables in the first block, $R^2 = .05$, $F(12, 410) = 1.93$, $p = .030$, but the addition of treatment description in the second block did not result in additional variance, $\Delta R^2 = .03$, $\Delta F(6, 404) = 1.84$, $p = .090$.

Lower age ($p = .001$) and income ($p = .020$), and full-time employment compared to unemployment ($p = .027$), were all associated with higher believability of the biological explanatory model. As for treatment description allocation effects, receiving a simple ($p \leq .001$) or complex ($p \leq .001$) biology focused description was associated with higher believability of the biological mechanistic model of CBT for depression than was receiving a generic description of

CBT mechanisms. In contrast, receiving a simple stress ($p = .005$) or simple relationship ($p = .041$) description was associated with lower believability in the biological explanatory model.

Among the demographic predictors, higher believability in the relationship-oriented explanatory model was associated with older age ($p = .030$), identifying as female compared to male ($p = .012$), and never employed compared to full-time employment ($p = .039$). Within the treatment description allocation block, receiving a complex biological description ($p = .050$) was associated with lower believability in the relationship oriented explanatory model than receiving a generic treatment description.

Table S1

Correlations Between Demographic Variables and CBT Believability

	Biological	Relationship	Stress
Age	-.15**	.08	.08
Income	-.09	-.03	-.06
Education	-.03	-.01	-.02
Gender (*n* = 424)	-.05	.10*	.10*
Marital status (*n* = 424)			
Currently dating	-.05	.02	.03
Single	.06	.05	.02
Divorced/Separated	.01	-.05	-.07
Married/Cohabiting	-.03	-.03	-.004
Employment			
Full-time	-.01	-.02	-.04
Part-time	.00	.03	.04
Unemployed	-.09	.02	.09
Not looking for work	.06	-.07	-.09
Never employed	.10*	.08	.06
Retired	-.001	-.02	-.02

Note. $N = 425$. One participant identifying as non-binary and one widowed participant were excluded from the analyses for gender and marital status respectively.

*$p \le .05$. **$p \le .01$

Table S2

Correlations Between Demographic Variables and scores on TAAS and CEQ

	TAAS	CEQ
Age	.03	-.04
Income	.13**	.09
Education	-.01	-.01
Gender (*n* = 448)	-.08	-.03
Marital status		
Dating	-.05	-.06
Single	-.07	-.03
Divorced/separated	-.08	.001
Married/cohabiting	.14**	.07
Widowed	.00	-.05
Employment (*n* = 448)		
Full-time	.11*	.03
Part-time	-.06	.06
Unemployed	-.004	-.05
Not looking for work	-.08	-.04
Retired	-.03	-.07

Note. N = 449. CEQ = Credibility/Expectancy Questionnaire. TAAS = Treatment Acceptability and Adherence Scale. One participant identifying as non-binary and one participant who had never been employed were excluded from the analyses for gender and employment respectively. *$p \leq .05$. **$p \leq .01$

Table S3

Summary of Hierarchical Regression for Biology Believability with Standardised Coefficients

	Block 1	Block 2
R^2	.07	.27
ΔR^2	.07	20.
ΔF	2.43**	18.86***
Age	-.18**	-.17**
Income	-.13*	-.13*
Education	.06	.08
Gender (1, female; 0, male)	-.05	-.07
Marital status (reference group: currently dating)		
Single/never married	.13	.002
Married/cohabiting	.14	.08
Separated/divorced	.11	.05
Employment (reference group: employed full-time)		
Employed part-time	-.04	-.01
Unemployed	-.12*	-.10*
Not looking for work	.02	.05
Never employed	.09	.08
Retired	.04	.001
CBT description (reference group: generic)		
Simple biology		.28***
Complex biology		.26***
Simple stress		-.16**
Complex stress		-.06
Simple relationship		-.12*
Complex relationship		-.05

Note. N = 423. Income and education levels were entered as ordinal variables.

* $p \leq .05$, ** $p \leq .01$, *** $p \leq .001$

Table S4

Summary of Hierarchical Regression for Relationship Believability with Standardised Coefficients

	Block 1	Block 2
R^2	.05	.08
ΔR^2	.05	.03
ΔF	1.64	2.35*
Age	.14*	.12*
Income	-.03	-.03
Education	.02	-.001
Gender (1, female; 0, male)	.12*	.13*
Marital status (reference group: currently dating)		
Single/never married	.02	.03
Married/cohabiting	-.06	-.05
Separated/divorced	-.10	-.07
Employment (reference group: employed full-time)		
Employed part-time	.005	.001
Unemployed	.007	-.002
Not looking for work	-.08	-.09
Never employed	.10	.10*
Retired	-.70	-.06
CBT description (reference group: generic)		
Simple biology		-.02
Complex biology		-.13*
Simple stress		-.01
Complex stress		-.09
Simple relationship		-.02
Complex relationship		.09

Note. N = 423. Income and education levels were entered as ordinal variables.

* $p \leq .05$, ** $p \leq .01$, *** $p \leq .001$

Table S5

Summary of Hierarchical Regression for Stress Believability with Standardised Coefficients

	Block 1	Block 2
R^2	.05	.08
ΔR^2	.05	.03
ΔF	1.93*	1.84
Age	.14*	.14*
Income	-.02	-.01
Education	.01	-.003
Gender (1, female; 0, male)	.12*	.11*
Marital status (reference group: currently dating)		
Single/never married	.-.02	.03
Married/cohabiting	-.06	-.09
Separated/divorced	-.11	-.05
Employment (reference group: employed full-time)		
Employed part-time	.02	.03
Unemployed	.08	.07
Not looking for work	-.09	-.07
Never employed	.08	.09
Retired	-.07	-.06
CBT description (reference group: generic)		
Simple biology		-.04
Complex biology		-.11
Simple stress		.06
Complex stress		.01
Simple relationship		-.10
Complex relationship		-.07

Note. N = 423. Income and education levels were entered as ordinal variables.

* $p \leq .05$, ** $p \leq .01$, *** $p \leq .001$
